# Functional similarity and competitive symmetry control productivity in mixtures of Mediterranean perennial grasses

**DOI:** 10.1371/journal.pone.0221667

**Published:** 2019-08-23

**Authors:** Luna Morcillo, Azucena Camacho-Garzón, Juan Sebastián Calderón, Susana Bautista

**Affiliations:** 1 Department of Ecology and IMEM, University of Alicante, Alicante, Spain; 2 Mediterranean Center for Environmental Studies (CEAM Foundation), Joint Research Unit University of Alicante-CEAM, University of Alicante, Alicante, Spain; Estacion Experimental de Zonas Aridas, SPAIN

## Abstract

Competition is a major factor structuring plant communities and controlling their productivity. The functional similarity between the interacting species and the context resource availability are assumed to be most critical factors that modulate the strength, sign, and outcome of plant competition, yet their roles and interactions are subjected to debate. In a glasshouse experiment, we constructed monocultures and bi-specific cultures of three common perennial grasses of Mediterranean drylands, the short grass *Brachypodium retusum* and the tussock grasses *Stipa tenacissima* and *Lygeum spartum*, and investigated how the functional similarity between these species modulate their interactions and culture productivity under contrasting levels of water availability. Regardless the degree of functional similarity between the interacting species, *B*. *retusum* consistently exhibited a greater competitive ability than the other two species, followed by *L*. *spartum*, and with *S*. *tenacissima* behaving as the weakest competitor. Bi-specific cultures of *B*. *retusum* and either *L*. *spartum* or *S*. *tenacissima* produced higher biomass than the average biomass of the respective monocultures (i.e. overyielding), whereas the combination of the most similar species, *L*. *spartum—S*. *tenacissima*, which exhibited the highest competition symmetry (i.e., the more similar mutual impact), did not show any significant overyielding. Higher water availability increased productivity and promoted transgressive overyielding for the most dissimilar species, *B*. *retusum* and *L*. *spartum*, which however exhibited intermediate competition asymmetry. This study calls attention to the thin line between differences in functional traits and competition asymmetry that could eventually lead to either competitive exclusion or resource partitioning and coexistence.

## Introduction

Competition has been since long considered the most significant interaction structuring plant communities [1–3] and thereby controlling ecosystem function [4]. Biotic factors such as the functional traits and similarity of the interacting species are known to affect the strength, sign, and outcome of plant-plant interactions [5, 6], yet the role of species functional similarity in shaping plant competition outcomes is still under debate [7, 8]. Understanding this role and how it can be modulated by the abiotic environment is critical for predicting how competition influences the structure and functioning of plant communities.

The ability of the species to compete with each other results from two components: the ability to suppress a neighbor (competitive effect) and the ability to avoid or resist being suppressed (competitive response), both jointly shaping the relative competitive performance of the interacting species [9, 10]. The competitive effect ability has been positively related to traits such as plant size, growth rate, and the production of allelopathic exudates, while the competitive response ability has been mostly associated with traits such as root development and seed size [10, 11]. The interaction between plants that strongly differ in traits related to their competitive abilities, either effect or response abilities, could result in a clear competitive hierarchy and a high degree of competition asymmetry (i.e., contrasting reciprocal impact), eventually leading to the suppression of the worst competitor [12]. However, increasing contrast in functional traits could also imply niche differentiation, and thereby reduced competition and higher probability of coexistence [13], particularly when niche differences exceed differences in the competitive ability of the interacting plants [7, 14].

If the species functional dissimilarity implies niche differentiation, more functionally diverse plant communities should be able to use resources more completely; reducing niche overlapping and resulting in a positive relationship between diversity and productivity through complementarity effect [15–19]. However, if the contrast of species traits is large, certain species or certain functional groups could have the favorable traits to provide the species with a greatest inherent productivity and competitive ability. This would support a composition effect on productivity, rather than diversity being the direct causing factor. This phenomenon is known as sampling effect [15, 20, 21] and can to lead to over-yielding (i.e. higher productive of diverse mixtures than the average productivity of the monocultures), but not to transgressive over-yielding (i.e. mixtures outperforming the best monoculture). Contrarily, complementarity effects would increase the net use of resources and improve the performance of the species in mixtures, potentially leading to transgressive over-yielding [22, 23].

Abiotic factors such as resource availability are recognized as modulators of the intensity of competition [24, 25], and therefore of the competition impacts on productivity of the community. However, the role played by resource availability in modulating competition is still an area of debate [5, 26–28]. While some authors have suggested that competition increases with increasing productivity [29–31], there is also evidence of the opposite pattern [32–34], as well as evidence of competition dominating at both ends of resource availability gradients [35, 36]. Further, little is known on how the availability of resources could influence the effects of biotic factors on competition. Direct manipulation of water availability, as the main limiting factor in drylands, can help to reveal the role of resource availability in generating fitness differences among co-occurring species and modulating the effects of functional similarity on resource competition.

To evaluate the effects of the functional similarity of the interacting plant species on the competition outcome and the productivity of the species mixture, and how these effects are modulated by the availability of resources, we analyzed interspecific versus intraspecific interaction effects and diversity effects on plant performance and culture biomass for three common grass species of Mediterranean drylands (*Brachypodium retusum* Pers., *Stipa tenacissima* L. and *Lygeum spartum* L.) under contrasting water availability. We hypothesized that (I) individuals in mono-specific cultures will experience stronger competition effects than in bi-specific cultures. (II) Plant-plant interaction outcome in bi-specific cultures will vary according to the functional similarity among species, with each species competing more intensely and symmetrically with the most functionally similar species. (III) Higher functional dissimilarity between the interacting species will result in higher probability of complementarity effects, leading to higher productivity relative to the respective monocultures. For the selected target species, this would imply higher biomass production for *B*. *retusum*–*L*. *spartum* cultures and lower production for *L*. *spartum*–*S*. *tenacissima* cultures. (IV) Higher water availability will increase the contrast in competitive ability between the species and the asymmetry of competition, as well as the probability of sampling effect in case of increased productivity in mixtures.

## Materials and methods

### Target species

We used three C_3_ perennial grass species that are common and abundant species in Mediterranean drylands: *Brachypodium retusum* (Pers.) P. Beauv., *Stipa tenacissima L*. *and Lygeum spartum L*. The three species belong to the *Poaceae* family, within the subfamily *Pooideae*. The phylogenetic analysis of the *Pooideae* group, [37, 38] supports the position of the *Lygeeae*, a tribe with a single species: *L*. *spartum*, as the second earliest diverging lineage in the subfamily. *Stipeae*, with 28 genera, appeared after two other lineages branched off, and *Brachypodieae* (20 species in one genus) appeared after one more diverging lineage, being the most recently evolved tribe of the three target groups. Regarding their morpho-functional traits, *S*. *tenacissima and L*. *spartum* form dense tussocks, with an average height of ~60 cm in the case of *L*. *spartum* and of ~90 cm in the case of *S*. *tenacissima*, while *B*. *retusum* is a short grass with a more extensive and erect growth, and stems of ~30 cm in height (S1 Appendix). The three species have an extensive below-ground rhizome, with rooting depths of 15–20 cm in *B*. *retusum*, and 30–40 in *S*. *tenacissima*, and *L*. *spartum*, respectively [39], yet the latter species can reach deeper depths [40]. The two tussock grasses are found in the driest environments of the Mediterranean basin and exhibit similar maximum photosynthetic rate and leaf nitrogen concentration, and leaf and canopy structures that help to reduce the impact of high-radiation environments. However, *L*. *spartum* seems to be adapted to more mesic conditions and saline soils than *S*. *tenacissima*, which has a more opportunistic growth and higher plasticity to respond to drought [41]. *Brachypodium retusum* has a wider geographical range and climate tolerance, being generally frequent in the Mediterranean basin, western Asia and Arabia, and growing in a wide variety of grassland and shrubland communities, from xerophilous grasslands and semiarid low matorral (tomillares) and steppes to tall maquis and garrigue in sub-humid areas [38, 42]. Various dissimilarity indices calculated from the available data on nine morpho-functional functional traits (S1 Appendix) consistently support that L. *spartum*—*S*. *tenacissima* (L-S) is the most similar species pair, followed by *B*. *retusum*—*S*. *tenacissima* (B-S), with an estimated dissimilarity of 1.4 times the L-S dissimilarity, and by the most dissimilar pair: *B*. *retusum*—*L*. *spartum* (B-L), with an estimated dissimilarity of 1.7 times the L-S dissimilarity (S1 Appendix).

### Experimental design

We prepared monospecific and bi-specific cultures of the target species in a glasshouse at the University of Alicante, Alicante (Spain). The experimental design included two main factors, culture type (monospecific and bi-specific) and watering regime (frequent and reduced watering). Bi-specific cultures consisted of all possible combinations of the three species (*B*. *retusum—L*. *spartum*, *B*. *retusum—S*. *tenacissima; and L*. *spartum—S*. *tenacissima*). Each combination of culture type (3 monospecific and 3 bi-specific) and watering regime was replicated 4 times, resulting in a total number of 48 cultures.

In July 2014, the cultures were established in plastic pots of 11x11 cm at the base and 16x16 cm at the top, and filled with a homogenized substrate up to 13 cm in height, resulting in a total volume of substrate of approximately 2.3 L. The substrate consisted of 40% of coconut fibre, 40% of red peat and 20% of fine silica sand (with particle size ranging between 0.2 and 0.7 mm), designed to guarantee a good drainage and to facilitate plant harvesting at the end of the experiment. We sowed 3 seeds per hole on 6 small holes per pot. The seeds were supplied by the Forest Seed Bank of the Valencian Forest Administration. During the first days of the experiment, we replaced non-germinating seeds and removed extra seedlings in case there was more than one seedling per hole, until we achieved the target density of one individual per sowing hole, and thus 6 individuals per pot, for all the cultures. The pots were randomly placed on a 1 x 3 m bench, and kept under natural daylight. To avoid possible local variation of light and other environmental factors, the relative location of the pots on the bench was changed weekly. Air temperature and relative humidity in the glasshouse varied between 19 and 31.5°C and between 65 and 75%, respectively, during the experiment. We applied 100 mL watering per pot (representing, approximately, an increase of 4% in soil moisture content) three times per week, for the frequent watering treatment (W++), and twice per week for the reduced watering treatment (W+). For the first 140 days, the cultures were monitored weekly for plant height and number of leaves of each individual. Data were averaged per pot. After 5 additional months, all pots were harvested; above and belowground plant material was separated at the stem base, and belowground material was delicately washed to remove soil remains attached to the roots. Above and belowground biomass was separately oven-dried for 72h at 80°C, and weighed afterwards.

### Data analyses

For each target species, we analyzed the average plant height and number of leaves per culture (pot) using a Repeated Measures analysis of variance, with Watering regime (W), with two levels, frequent watering (W++) and reduced watering (W+), and accompanying Species (S), with three levels (B, L and S) as between-subject factor, and Time (T) as within-subject factor. Biomass data (above-ground biomass, below-ground biomass, total biomass) were analyzed by using a Mixed-effects analysis of variance with two fixed factors: watering (W), with two levels, (frequent watering (W++) and reduced watering (W+)) and Culture (C), with two levels (mono and bi-specific), and one random factor: Species combination (SC), with six levels (B-B, L-L, S-S, B-L, B-S, L-S). For each target species, we calculated the net interspecific competition effect of each competing species on final plant height and number of leaves as the difference between the average values for the plants growing in each bi-specific culture and the average values for the monospecific cultures of the target species. From these values and adapting the approach proposed by Johansson and Keddy [12], we estimated pairwise interspecific competition asymmetry for species *i* and *j* as the absolute difference between the net interspecific competition effects (NE) of the two species on each other (|NE_i-j_—NE_j-i_|), with higher values indicating higher asymmetry. We estimated overyielding and transgressive overyielding effects on aboveground, belowground and total biomass as the difference between the biomass produced by each bi-specific culture and either the average of the respective monospecific cultures (i.e. overyielding) or the respective most productive monoculture (i.e transgressive overyielding). We evaluated the statistical significance of the net interaction effects on plant height and number of leaves, and the significance of the overyielding and transgressive overyielding as significant deviations from zero using two-tailed Student’s one-sample t-tests. All data met the normal distribution of residuals and homoscedasticity assumptions. All statistical analyses were performed by using v.23.0 Statistical package (SPSS Inc., Chicago, IL, USA).

## Results

### Accompanying species and watering effects on individual plant growth

**Fig 1:**
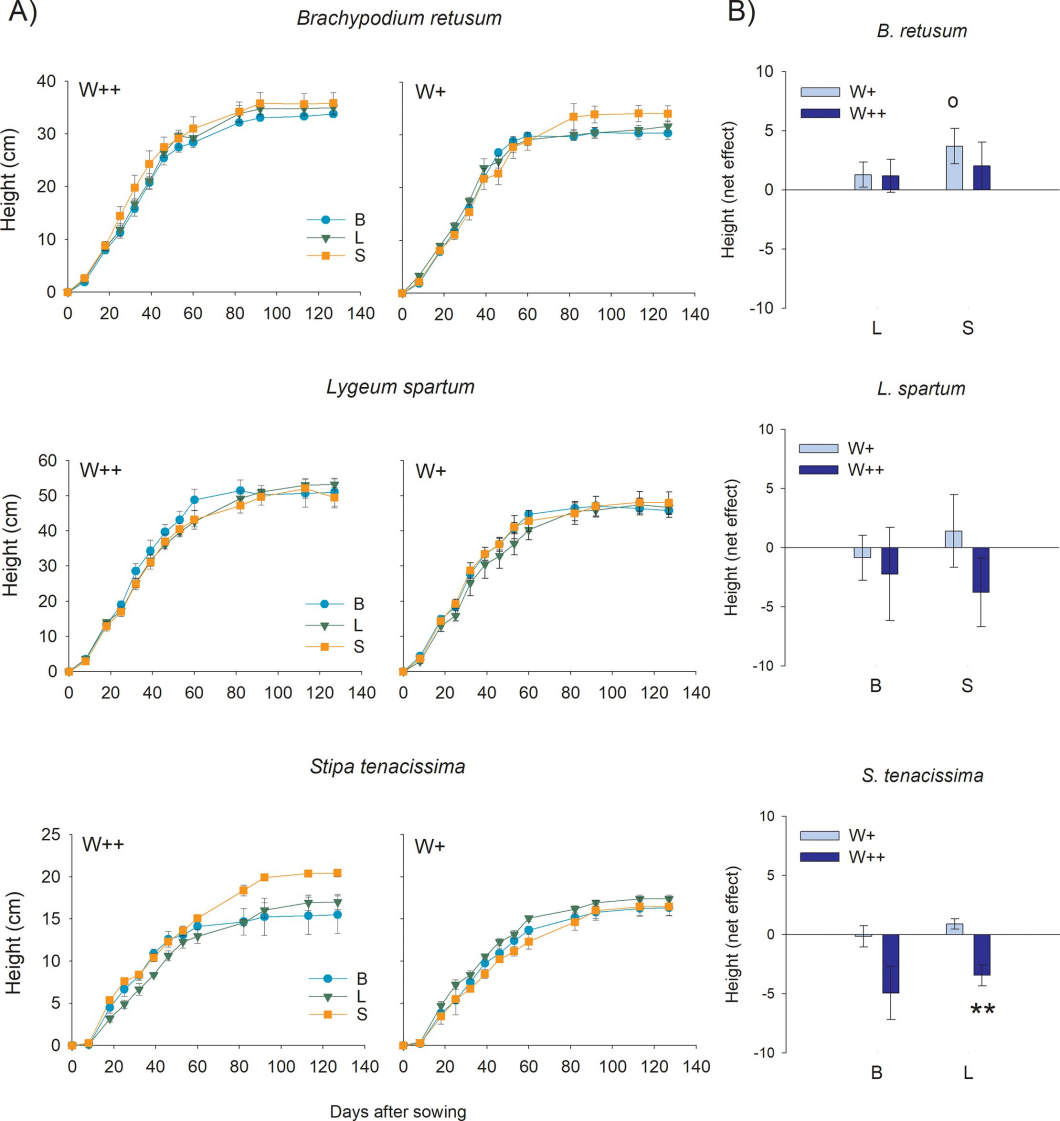
Pairwise interaction effect on plant height. A) Average (±1 SE) height for the three target species as a function of the accompanying species: B (*B*. *retusum*, light blue circles); L *(L*. *spartum*, green triangles); S (*S*. *tenacissima*, orange squares) and the watering regime applied (W++, frequent watering; W+, reduced watering). B) Difference (net effect) in final plant height at the end of the study period (140 days) between each bi-specific culture and the average of the respective monospecific cultures for each target species. Asterisks and empty circles represent, respectively, significant and marginally significant deviations of net effects from zero (two-tailed Student’s one-sample t-tests): p<0.01 (**), and p<0.06 (°); n = 4. Note the change in Y axis scaling.

The time for seedling emergence was very similar for *L*. *spartum* and *B*. *retusum* (around 6 days after sowing), and slightly delayed for *S*. *tenacissima* (around 11 days after sowing). Plant height increased quickly during the first two months, and then tended to a plateau, with values around 50, 40 and 20 cm for *L*. *spartum*, *B*. *retusum* and *S*. *tenacissima*, respectively. *Stipa tenacissima* showed the lowest height growth rates (Fig 1, panel A). The growth in height did not significantly vary with either watering treatment or accompanying species, yet there were significant interactions between the two factors and time (T x W x S) and between watering and time (T x W) for some of the species (Table 1). For *S*. *tenacissima*, there was a significant interaction between watering and accompanying species (W x S), with individuals of this species showing higher height in monocultures than in bi-specific cultures in case of frequent watering and the opposite pattern (lower height in monocultures) under reduced watering. The difference in final height between the plants growing in the bi-specific cultures and in the respective monospecific cultures (net interspecific competition effect) did not significantly vary from zero (Fig 1, panel B), except for *S*. *tenacissima* under frequent watering, which showed a negative effect of the interaction with *L*. *spartum*, and for *B*. *retusum* under reduced watering, which showed a positive effect of the interaction with *S*. *tenacissima*, as compared with the respective intraspecific interaction effect. Thus, the competitive effect of *L*. *spartum* was higher on *S*. *tenacissima* than on *B*. *retusum*, and the competitive response of *B*. *retusum* was stronger against *S*. *tenacissima* than against *L*. *spartum*.

**Table 1:** Analysis of treatment effects on plant height and number of leaves for the three target species.

	*B*. *retusum*		*L*. *spartum*		*S*. *tenacissima*	
	Height	Number of leaves	Height	Number of leaves	Height	Number of leaves
**T** df:11/7	**990.9 (<0.001)**	**271.3 (<0.001)**	**968.1 (<0.001)**	**58.5 (<0.001)**	**518.5 (<0.001)**	**86.1 (<0.001)**
**W** df: 1	2.3 (0.144)	*4*.*3 (0*.*054)*	1.4 (0.251)	**19.3 (<0.001)**	1.3 (0.275)	1.9 (0.186)
**S** df: 2	1.1 (0.362)	**13.9 (<0.001)**	0.5 (0.636)	**5.5 (0.014)**	0.7 (0.505)	**21.8 (<0.001)**
**W x S** df: 2	0.4 (0.696)	0.3 (0.725)	0.4 (0.705)	0.1(0.879)	**6.0 (0.010)**	0.6 (0.547)
**T x W** df: 11/7	**3.3 (<0.001)**	**5.7 (<0.001)**	**4.3 (<0.001)**	**12.8 (<0.001)**	0.4 (0.950)	0.6 (0.710)
**T x S** df: 22/14	1.4 (0.112)	**6.1 (<0.001)**	**1.8 (0.016)**	**2.3 (0.009)**	**2.4 (0.001)**	**17.0 (<0.001)**
**T x W x S** df: 22/14	**2.3 (0.002)**	0.4 (0.981)	0.3 (0.998)	0.3 (0.992)	**1.7 (0.032)**	1.1 (0.379)

Values are F (P value) calculated using Repeated Measures Analysis of Variance; Time (T) is within-subject factor; Watering regime (W) and accompanying species (S) are between-subject factors; df: degrees of freedom. Numbers in bold highlight significant (p<0.05) effects; numbers in italics highlight marginally significant effects (p<0.1).

**Fig 2:**
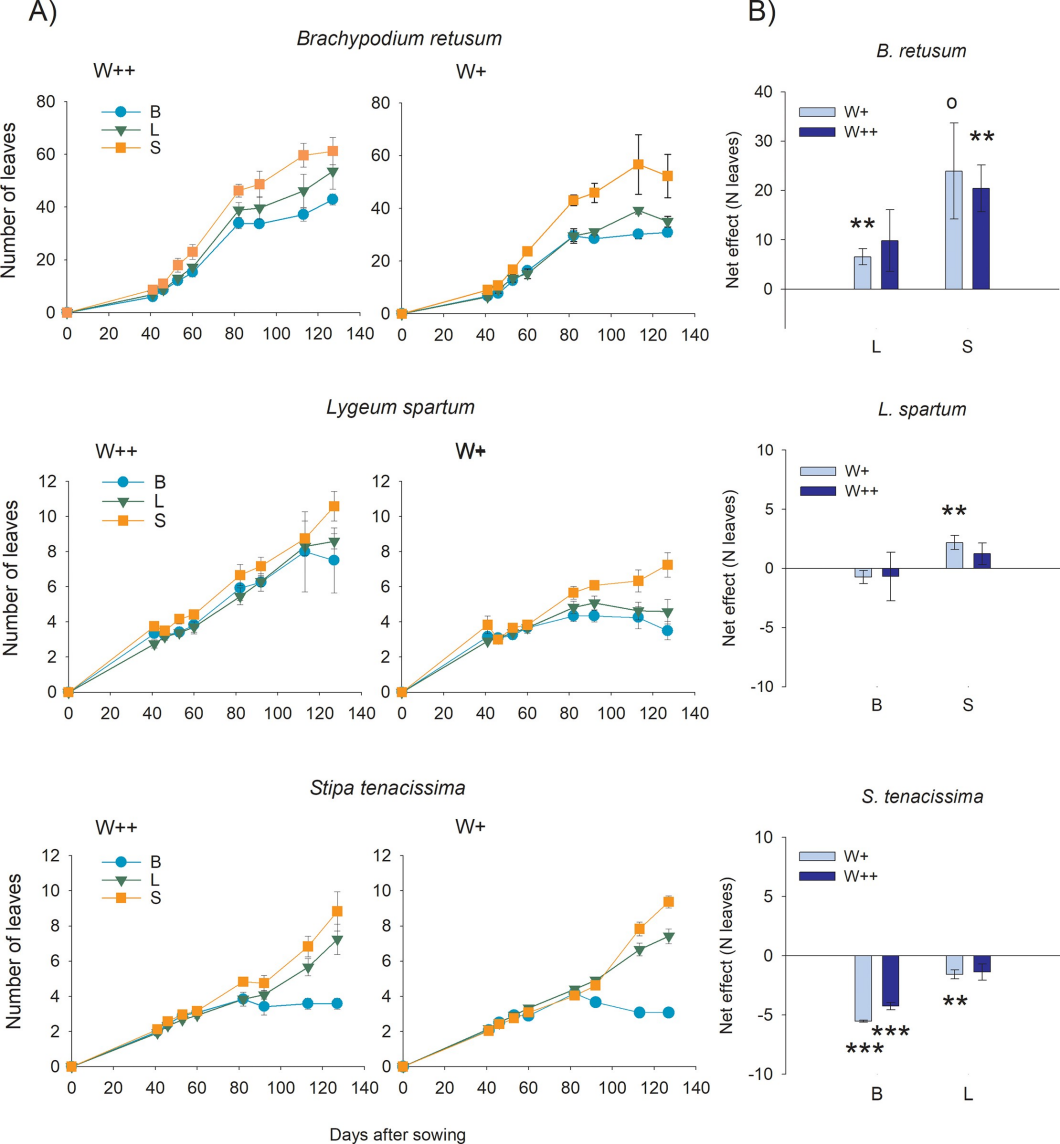
Pairwise interaction effect on the plant number of leaves. A) Average (±1 SE) number of leaves for the three target species as a function of the accompanying species: B (*B*. *retusum*, light blue circles); L (*L*. *spartum*, green triangles); S (*S*. *tenacissima*, orange squares) and the watering regime applied (W++, frequent watering; W+, reduced watering). B) Difference (net effect) in the number of leaves per plant between the bi-specific cultures and the average of the respective monospecific cultures, for the whole studied period (140 days) for each target species. Asterisks and empty circles represent, respectively, significant and marginally significant deviations of net effects from zero (two-tailed Student’s one-sample t-tests): p<0.001 (***); p<0.01 (**), p<0.05 (*), and p<0.06 (°); n = 4. Note the change in Y axis scaling.

Between-treatment differences in the number of leaves started to be noticeable around 80 days after sowing, once growth in height slowed down. Two of the target species, *B*. *retusum* and *L*. *spartum*, showed higher number of leaves under the frequent watering treatment (Fig 2, panel A; Table 1). For the three species, the number of leaves significantly varied with the accompanying species, being higher in cultures that included *S*. *tenacissima* and lower in cultures that included *B*. *retusum* (Table 1). The comparison of the pairwise net interspecific competition effects (i.e., relative to the respective intraspecific competition effect of each target species) on the number of leaves (Fig 2, panel B) showed a significant negative impact of *B*. *retusum* on *S*. *tenacissima*, and no effect on *L*. *spartum*; a negative impact of *L*. *spartum* on *S*. *tenacissima* under frequent watering, and no effect on *B*. *retusum*; and a higher competition effect of *S*. *tenacissima* on *L*. *spartum* than on *B*. *retusum*. Looking at the competition response against the interacting species, the difference in the final number of leaves between bi-specific and monospecific cultures of *B*. *retusum*, showed a positive effect of the interaction with either *L*. *spartum* or *S*. *tenacissima* as compared with the intraspecific interaction effect, yet this effect was lower (i.e., weaker competitive response) for the interaction with *L*. *spartum*. For *L*. *spartum*, there were no significant differences between interspecific and intraspecific interaction effects on the number of leaves, except for a significant positive effect of the interaction with *S*. *tenacissima* under reduced watering (i.e., stronger competitive response against *S*. *tenacissima* than against *B*. *retusum* and against conspecific individuals). For *S*. *tenacissima*, we found a negative effect of the interaction with any of the two other species as compared with the intraspecific interaction effect, with a weaker interspecific competitive response under reduced watering and particularly weak against *B*. *retusum* (Fig 2, panel B).

The species pair *B*. *retusum*–*S*. *tenacissima* exhibited the largest interspecific competition asymmetry, followed by *B*. *retusum*–*L*. *spartum*, and with *L*. *spartum*–*S*. *tenacissima* showing the most symmetrical interspecific competition. The interspecific competition asymmetry and the asymmetry gradient between the species pairs were clearer for the number of leaves than for plant height values (Table 2). There was a trend towards increased asymmetry with increased water availability for the *B*. *retusum*–*L*. *spartum* pair, and towards the opposite trend (increased asymmetry with decreased water availability) for the *L*. *spartum*–*S*. *tenacissima* pair; competition asymmetry for *B*. *retusum*–*S*. *tenacissima* did not show any clear response to water availability.

**Table 2:** Competition asymmetry on plant height and number of leaves for the three pairs of interacting species.

Interacting species pair	Height		Number of leaves	
	W+	W++	W+	W++
**B-L**	2.2 ± 2.2	3.4 ± 4.2	7.3 ± 1.7	10.5 ± 6.6
**B-S**	3.9 ± 1.8	7.0 ± 3.0	29.5 ± 9.7	24.7 ± 4.8
**L-S**	0.5 ± 3.1	0.3 ± 3.0	3.8 ± 0.7	2.6 ± 1.1

Asymmetry values estimated from the average (± SE) net interspecific competition effects of species *i* on species *j* (NE*ij*) and of species *j* on species *i* (NE*ji*) on the plant number of leaves (Fig 2, panel B) as |NE_i-j_—NE_j-i_|; SE = (SE_NE*ij*_^2^ + SE_NE*ji*_^2^)^1/2^. B, L and S represent *B*. *retusum*, *L*. *spartum*, and *S*. *tenacissima*, respectively.

### Species combination and watering effects on culture biomass

**Fig 3:**
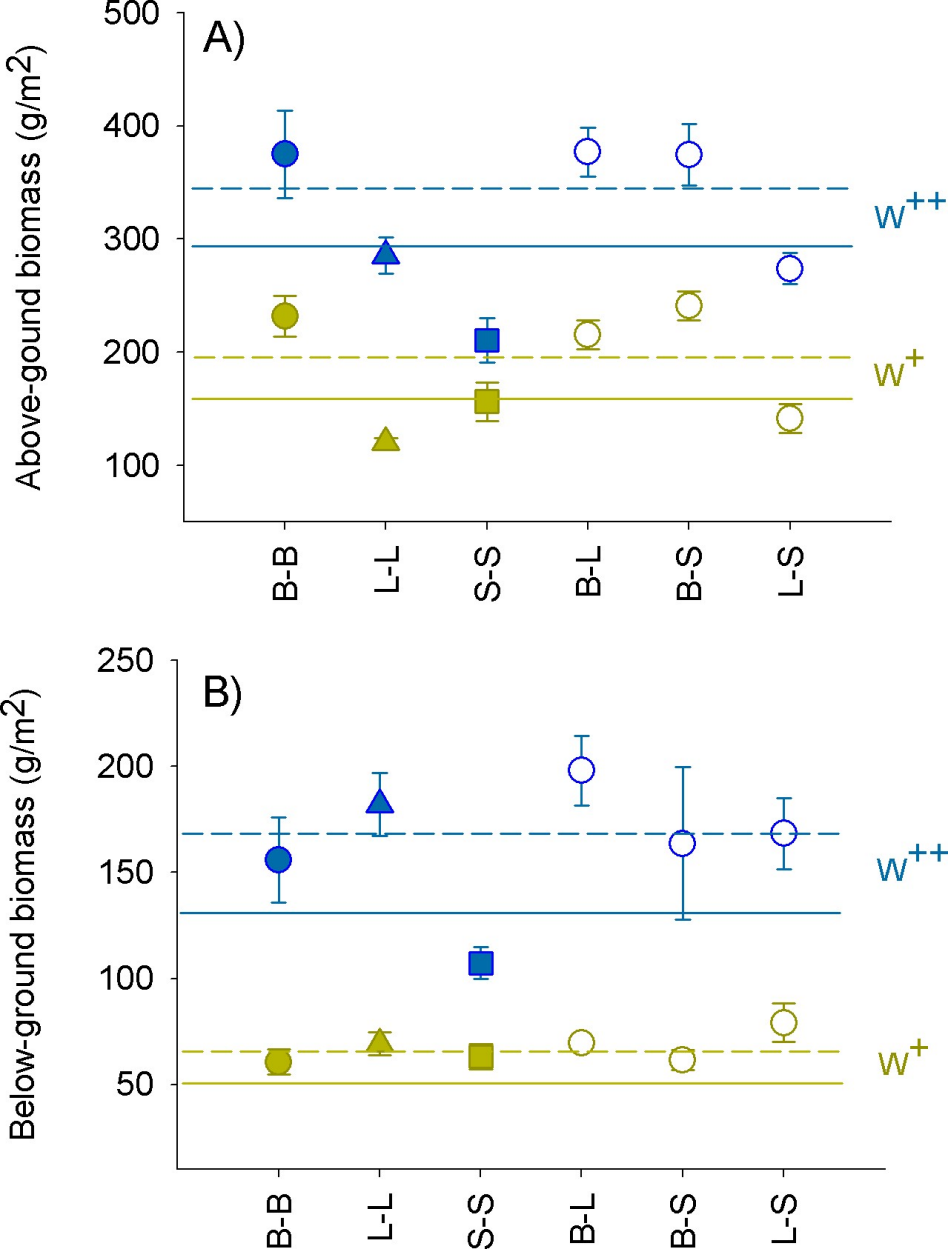
Culture biomass as a function of species combination and watering. Above-ground (A) and below-ground (B) biomass (average ± 1 SE; n = 4). B-B, L-L, and S-S: monocultures of *B*. *retusum*, *L*. *spartum* and *S*. *tenacissima*, respectively; B-L, B-S, and L-S: bi-specific cultures of each pair of species, with B, L and S representing *B*. *retusum*, *L*. *spartum*, and *S*. *tenacissima*, respectively. Solid line: average biomass for monospecific cultures; dashed line: average biomass for bi-specific cultures.

Culture biomass was significantly higher under frequent watering than under reduced watering (Table 2), with the largest differences between watering regimes for the combination of *B*. *retusum* and *L*. *spartum* (B-L), and the smallest difference for the monoculture of *S*. *tenacissima* (Fig 3). For monospecific cultures and frequent watering, there was a clear productivity trend from *B*. *retusum*, which produced the highest above-ground biomass, to *S*. *tenacissima*, which produced the lowest biomass. Below-ground biomass showed a different trend, with *L*. *spartum* producing the highest biomass, followed by *B*. *retusum*. Under reduced watering *L*. *spartum* produced the lowest above-ground biomass (Fig 3). For bi-specific cultures, the two combinations including *B*. *retusum* (B-L and B-S) showed the highest biomass (Fig 3). In general, both above and below-ground biomass in bi-specific cultures tended to be higher than in monospecific cultures, particularly under frequent watering (Fig 3), yet neither the culture (mono vs bi-specific) effect or the interaction between culture and watering were significant (Table 3). The different specific combinations (B-B, L-L, S-S, B-L, B-S, and L-S) significantly varied in above-ground biomass, but differences in below-ground and total biomass were not significant. Total biomass and above-ground biomass showed, respectively, a significant and marginally significant interaction between species combination and watering (Table 3).

**Table 3:** Analysis of treatment effects on total, above-ground and below-ground biomass.

	Total biomass	Above-ground biomass	Below-ground biomass
**W** df: 1	**60.4 (0.001)**	**55.8 (0.002)**	**64.9 (0.001)**
**C** df: 1	1.2 (0.330)	0.7 (0.451)	1.6 (0.268)
**SC** df: 4	3.2 (0.145)	**7.7 (0.037)**	1.3 (0.415)
**W x C** df: 1	0.6 (0.492)	0.4 (0.572)	0.9 (0.396)
**W x SC** df: 4	**3.7 (0.013)**	*2*.*4 (0*.*068)*	1.8 (0.141)

Values are F (P value) calculated using Mixed-effects ANOVA Analysis of Variance; Watering (W) and Culture (C) are fixed factors; Species Combination (SC) is a random factor; df: degrees of freedom. Numbers in bold highlight significant (p<0.05) effects; numbers in italics highlight marginally significant effects (p<0.1).

**Fig 4:**
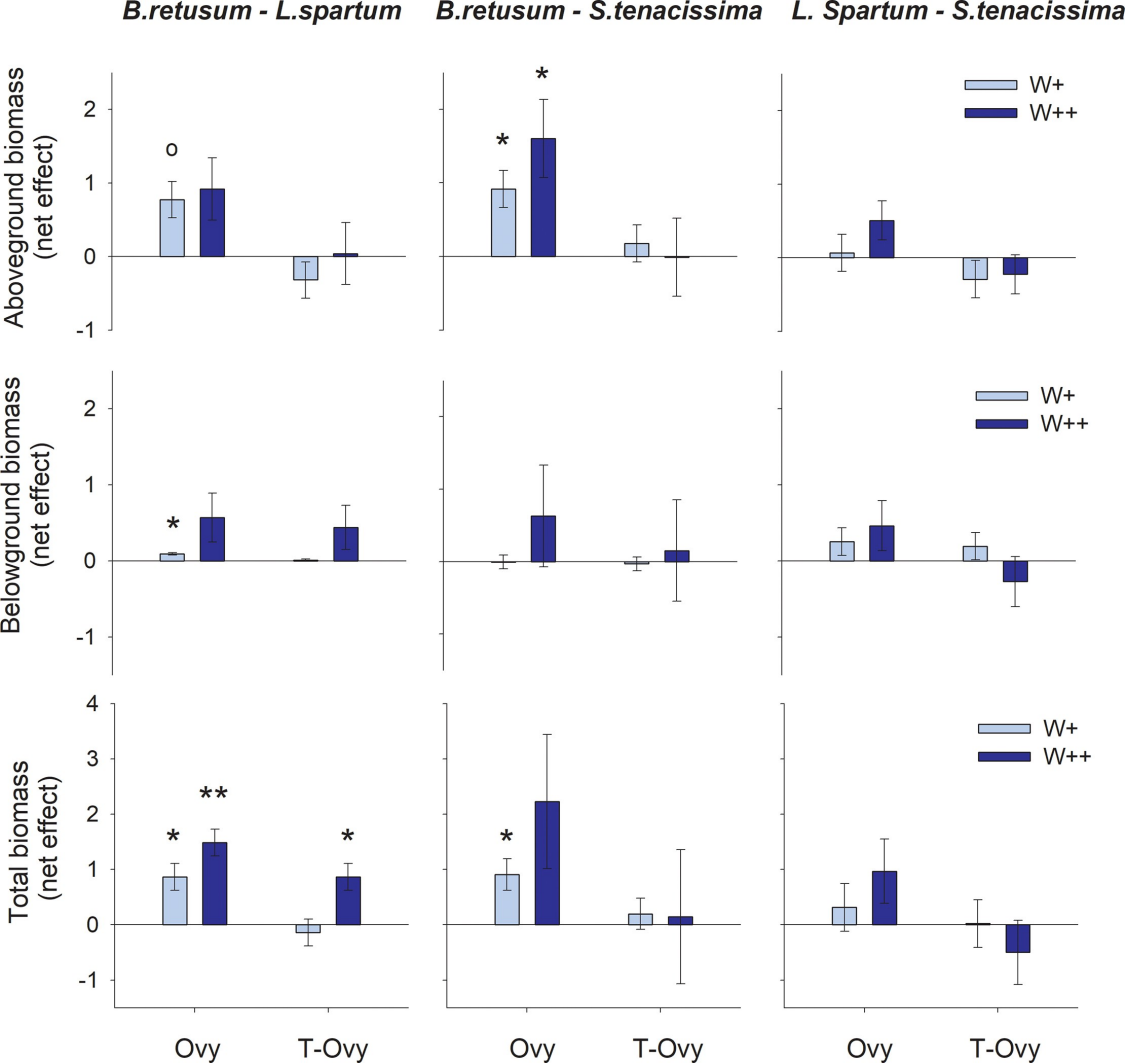
Net effect of species combination on culture biomass. Differences (net effect) in aboveground biomass (upper panel), belowground biomass (middle panel) and total biomass (lower panel) per pot (g) between the three target bi-specific cultures (*B*. *retusum—L*. *spartum*; *B*. *retusum—S*. *tenacissima*; *L*. *spartum—S*. *tenacissima*) and either the average of the respective monospecific cultures, i.e overyielding effect (Ovy), or the respective most productive monoculture, i.e transgressive overyielding (T-Ovy), for both frequent (W++) and reduced (W+) watering. Asterisks and empty circles represent, respectively, significant and marginally significant deviations of net effects from zero (two-tailed Student’s one-sample t-tests): p<0.01 (**), p<0.05 (*), and p<0.06 (°).

In general, bi-specific cultures of *B*. *retusum* and either *L*. *spartum* or *S*. *tenacissima* produced higher biomass than the average biomass of the respective monocultures (i.e. overyielding effect), with this effect being slightly more pronounced under frequent watering and for the combination *B*. *retusum—S*. *tenacissima* (B-S). The combination *L*. *spartum—S*. *tenacissima* (L-S) did not show any significant overyielding. When compared with the best monoculture of each combination, bi-specific cultures performed better only for the couple *B*. *retusum—L*. *spartum* (B-L) under frequent watering, which produced significantly higher biomass than *B*. *retusum* monocultures (i.e., transgressive overyielding). In two cases (B-L and L-S aboveground biomass under reduced watering) comparison with the respective best monoculture pointed to underyielding, yet this effect was not significant (Fig 4).

## Discussion

We examined how the functional similarity between plant species modulate their interactions and overall culture productivity for three common perennial grass species in Mediterranean drylands, and whether these effects varied depending on water availability. We did not find a simple, monotonic relationship between the degree of functional similarity and the competition strength and symmetry between each pair of target species. The contrast in morpho-functional traits between the target species did not systematically entail niche differentiation and reduced competition, but rather a greater competitive ability of *B*. *retusum*, followed by *L*. *spartum*, and with *S*. *tenacissima* as the weakest competitor. Our results suggest, however, that both sampling and complementarity effects, attributable to the interaction between species with contrasting traits, could have contributed in a combined way to the positive effect of diversity in the productivity of the cultures. Water availability modulated the competitive asymmetry between species and thus the productivity of the species mixtures.

### Functional similarity as modulator of competition between grass species

Regardless the functional similarity between each pair of target species, any of the species performed better when growing with *S*. *tenacissima* and worse when growing with *B*. *retusum*, indicating a lower and higher competitive ability of these two species, respectively. Both the competitive effect and the competitive response abilities [9] responded in a broadly consistent way to the functional differences between the interacting species. Thus, for any of the target species, the interspecific competitive effect was higher on the most similar species, and the interspecific competitive response ability was stronger against the most similar species. However, depending on the target species considered, the intraspecific competitive response ability was either stronger or weaker than the interspecific competitive response. Previous studies have reported both positive correlations and no correlation between competitive effect and response hierarchies among species [43, 44], and the potential functional links between these two forms of competition are still unclear [10, 11]. The different competitive ability rank of the interacting species resulted in a gradient of competition asymmetry for the species pairs that did not fully correspond with their functional dissimilarity. Thus, the most asymmetrical pair was *B*. *retusum–S*. *tenacissima*, which exhibited intermediate dissimilarity, while the most functionally dissimilar *B*. *retusum–L*. *spartum* pair showed intermediate competition asymmetry, as compared with the other two target pairs. These results differ from our initial hypothesis, according to which we expected increased competition strength and symmetry with increasing morfo-functional similarity between the interacting species, and point to the importance of the hierarchy in competitive ability, and the plant traits that explain such a hierarchy, as a control factor of competition asymmetry and thus of the overall competition outcome.

*Brachypodium retusum* largely differs in its morpho-functional traits from the two other species. While *L*. *spartum* and *S*. *tenacissima* are characterized by low vegetative colonization ability (very short, almost inexistent spacing between ramets) and a dense tussock growth form (phalanx strategy, *sensu* [45]), *B*. *retusum* exhibits an intermediate strategy between phalanx and guerrilla [46] with longer spacing and higher capacity for colonizing space. These contrasting growth forms can largely affect the competitive ability of clonal species [47]. Under a relatively low biomass of neighbors, as the initial condition of our experiment, the higher colonization ability of *B*. *retusum* would confer a competitive advantage to this species as compared with the other two. Furthermore, *B*. *retusum* was the most productive species in the experiment, which must have contributed to a high level of relative crowding (sensu [48]) of *B*. *retusum* in bi-specific cultures, and therefore to a larger competitive ability, as compared with the two tussock grasses. Despite *S*. *tenacissima* and *L*. *spartum* are morphologically quite similar, there are also some important trait differences between them. For example, *S*. *tenacissima* exhibits a more plastic response to environmental changes, resistance to drought, and opportunistic growth than *L*. *spartum*, while *L*. *spartum* seems to be adapted to less dry conditions and more saline and nutrient-poor soils [41, 49]. These differences explain that these two species often appear segregated in space within the same community in response of microsite variation, yet they both coexist for a wide range of environmental conditions [41]. Under the conditions of the experiments, with relative water scarcity under reduced watering and no stress due to soil salinity, the main trait differences between the two tussock species that may have led to the higher competitive ability of *L*. *spartum* as compared *with S*. *tenacissima* are probably related to the potential for a larger and deeper root system [10, 50], such as higher rooting depth and root width in *L*. *spartum* (S1 Appendix).

The large morpho-functional contrast between the short grass *B*. *retusum* and the two (similar) tussock grasses did not appear to drive the pairwise competition outcome. Lifeform-independent traits that favored either above-ground (*B*. *retusum*) or below-ground (*L*. *spartum*) productivity under the experimental conditions seem to have played the most relevant role in modulating their competitive ability hierarchy and competition outcomes. Thus, while SLA is much larger for *B*. *retusum* than for *L*. *spartum*, rooting depth and root diameter are much larger for *L*. *spartum* than for *B*. *retusum* (S1 Appendix). These results highlight the importance of trait specific and context specific contributions to the overall variance in the relative competitive performance of the interacting species. In this regard, the timing of seed germination could be of particular relevance, as very short delays in seedling emergence can entail high differences in final biomass and reproduction, especially under competitive conditions [51–53]. For instance, when interspecific competition for light is intense as seedling density increases, early emergence and establishment might be critical [54]. In our experiment, *S*. *tenacissima* germinated slightly later and grew slightly slower than *B*. *retusum* and *L*. *spartum*, which appeared to have consequences in its competitive ability.

### Effects of pair-wise species combinations on culture biomass

Comparison of the performance of species mixtures with monocultures is an essential tool in the evaluation of biodiversity effects [55]. We found overyielding (i.e. higher biomass for the species mixtures than the average biomass of the respective monocultures) for the cultures that combined the short grass *B*. *retusum* with any of the two tussock grasses, whereas the combination *L*. *spartum—S*. *tenacissima*, which exhibited the lowest competition asymmetry and the highest morpho-functional similarity, did not show any significant overyielding. The observed pattern of overyielding, associated to the higher inherent productivity of *B*. *retusum*, pointed to sampling effect as the main mechanism underlying a higher productivity in bi-specific cultures [55]. This suggests that functional contrast between the species mostly resulted in the advantage of the best competitor. However, we found transgressive overyielding (bi-specific culture performing better than the best monoculture of the combination) in the case of *B*. *retusum—L*. *spartum* under frequent watering, which suggests improved resource use through complementarity [56, 57]. The fact that these species showed contrasting growth patterns, with *B*. *retusum* producing higher above-ground and lower below-ground biomass than *L*. *spartum* in their respective monocultures, could explain a complementary resource use. This complementary use would have fully operated under frequent watering, which particularly promoted root growth in *L*. *spartum*. It could also explain that this species combination exhibited less competition asymmetry than the highly asymmetric *B*. *retusum*–*S*. *tenacissima* pair, as the respective dominance of the aboveground and belowground compartments by *B*. *retusum* and *L*. *spartum* could have partly counterbalanced the morpho-functional differences between short-grass and tussock-grass life forms. Strong facilitative interactions may be required to generate consistent transgressive overyielding and overall positive effects of diversity on productivity [56, 58]. However, we found a neutral effect of *B*. *retusum* on *L*. *spartum* performance, and just a slightly positive effect of *L*. *spartum* on the number of leaves of *B*. *retusum* as compared with the effects of equal density of conspecifics, which supports resource partitioning over facilitation as the mechanism driving the observed complementarity effect [59, 60]. The combinations including the two tussock grasses were the least productive, exhibiting no overyielding and quite symmetrical competition between the two species, despite the delay in the germination of *S*. *tenacissima*. Given the apparently small niche differentiation between these two species, their coexistence would also require small competitive asymmetry [7]. Armas and Pugnaire [61] found no differences between intraspecific and interspecific competition with *L*. *spartum* on *S*. *tenacissima* biomass under abundant resource supply, while competition of *S*. *tenacissima* was stronger than intraspecific competition for *L*. *spartum*. Under reduced watering, we found the opposite trend, yet in both studies differences between intra and interspecific competition for these two species were small. Overall, the results suggest that relatively small variations in the competitive asymmetry of the interacting species could shift the outcome of plant-plant interactions from resource partitioning, associated to intermediate asymmetries, to dominance and competitive exclusion when asymmetries are strong enough.

### The effect of water availability on the competition outcome

Water availability was crucial for the productivity of the cultures, which overall doubled yield from reduced to frequent watering. This indicates that water was limiting under the experimental conditions, even more as plants aged, as watering treatments were not changed throughout the study period, and therefore the share of water per biomass unit decreased with time. Water availability also changed the relative order of culture productivity. Thus, while monocultures of *S*. *tenacissima* were less productive than monocultures of *L*. *spartum* under frequent watering, the opposite pattern was found for the most stressful conditions of reduced watering. Similarly, the highest belowground biomass was produced by *B*. *retusum–L*. *spartum* under frequent watering and by *B*. *retusum–S*. *tenacissima* under reduced watering. These changes may reflect the higher resistance to drought and opportunistic growth of *S*. *tenacissima*, better suited than *L*. *spartum* to use pulses of resources in water-stressed environments [41].

Abiotic stress due to limiting resource availability is expected to modify the sign and intensity of plant-plant interactions, yet the direction of the changes is still unclear [5, 62–64] In our case, the effects of manipulating water availability were not consistent across pairwise species combinations and variables measured. For example, higher water availability increased the negative impact of *L*. *spartum* on *S*. *tenacissima* height, but reduced the negative effect of *B*. *retusum* and *L*. *spartum* on *S*. *tenacissima* number of leaves. Our results neither support nor contradict that competitive interactions increase in strength under more benign conditions [5]. They highlight, however, that changes in resource availability can drive shifts from resource partitioning to dominance. Thus, bispecific cultures of *B*. *retusum* and *L*. *spartum* exhibited transgressive overyielding for frequent watering and non-transgressive overyielding for reduced watering, indicating that complementarity in resource use was enhanced by increased water availability.

## Conclusions

For three common Mediterranean perennial grasses, we found a clear competitive ability hierarchy that in turn resulted in a gradient of pair-wise competitive asymmetry, with *B*. *retusum* and *S*. *tenacissima* being the best and worst competitor, respectively, and the combination of the two tussock grasses *S*. *tenacissima* and *L*. *spartum* being the most symmetrical pair in terms of competitive ability. The strength of pairwise competition and the degree of competitive asymmetry did not fully correspond with the morpho-functional dissimilarity of the interacting species, as the species did not always compete more intensely and symmetrically with the most functionally similar neighbor species. However, both morpho-functional dissimilarity and competitive asymmetry could have jointly determined the productivity of the species mixtures. While the most functionally similar and competitively symmetrical pair *S*. *tenacissima* and *L*. *spartum* did not result in overyielding, the most dissimilar yet moderately asymmetrical *B*. *retusum*–*L*. *spartum* pair resulted in transgressive overyielding through complementarity, and the moderately dissimilar yet highly asymmetrical *B*. *retusum* and *S*. *tenacissima* pair increased productivity through the dominance of *B*. *retusum*. Water availability modulated the outcome of pairwise interactions, with higher water supply enhancing productivity and increasing the probability of either transgressive or non-transgressive overyielding, depending on the species combination. Our results suggest that small variations in the functional traits and the conditions that modulate the competitive ability of the species could shift the interaction outcome and eventually lead to either dominance or resource partitioning, which adds plasticity and overall variance to plant-plant interactions.

## Supporting information

S1 Appendix Pair-wise trait-based functional dissimilarity. Pair-wise functional dissimilarity between the grass species *Brachypodium retusum*, *Stipa tenacissima* and *Lygeum spartum* estimated from available data on a variety of morpho-functional traits; includes mean value (and standard deviation), and references for the traits used to calculate the pairwise functional dissimilarity.
(DOCX)

S1 Dataset Supporting data. Mean and standard error values for height, number of leaves, and biomass data for the target species and culture types and treatment combination.
(XLSX)
