# Supplementary material for: Functional similarity and competitive symmetry control productivity in mixtures of Mediterranean perennial grasses
Source: PLoS One. 2019 Aug 23;14(8):e0221667. doi: 10.1371/journal.pone.0221667 (PMC6707634; doi:10.1371/journal.pone.0221667)
Supplement: S1 Appendix — Pair-wise functional dissimilarity between the grass species Brachypodium retusum, Stipa tenacissima and Lygeum spartum estimated from available data on a variety of morpho-functional traits; includes mean value (and standard deviation), and references for the traits used to calculate the pairwise functional dissimilarity. (DOCX) [file pone.0221667.s001.docx]

**S1-APPENDIX**

**Pair-wise trait-based functional dissimilarity between *Brachypodium retusum*, *Stipa tenacissima* and *Lygeum spartum***

Luna Morcillo^1,2^, Azucena Camacho-Garzón^1^, Juan Sebastián Calderón^1^, Susana Bautista^1^.

^1^ Department of Ecology and IMEM, University of Alicante, Alicante, Spain

^2^ Mediterranean Center for Environmental Studies (CEAM Foundation), Joint Research Unit University of Alicante-CEAM, University of Alicante, Alicante, Spain.

We estimated the pair-wise functional dissimilarity between the grass species *Brachypodium retusum*, *Stipa tenacissima* and *Lygeum spartum* by calculating the Euclidian distance, the Gower metric, and the Bray-Curtis dissimilarity index [1, 2, 3 4] from the available data on a variety of morpho-functional traits (Table S1). For each trait considered, we used the average value of all the values reported in the literature. To facilitate the combination of different trait data in the calculation of dissimilarity indexes, we rescaled the units of the trait values so that they all varied within similar ranges of magnitude. Furthermore, we also considered the potential effect of prior data standardization, and calculated the indexes using unstandardized trait data, Ln-transformed trait data, and trait values relative to the maximum value for the target species. The latter transformation was not applied to the Gower metric, as this index already considers relative differences in the trait values. The Euclidean distance was calculated as: $ED=\sqrt{\sum_{i} {(Xia-Xib)}^{2}}$, the Gower metric as: $GD=\frac{1}{N}\sum_{i=1}^{N} \frac{\left| Xia-Xib \right|}{\max\left( Xi \right)-min(Xi)}$, and the Bray-Curtis dissimilarity index as: $BC=\frac{\sum_{i} \left| Xia-Xib \right|}{\sum_{i} \left( Xia+Xib \right)}$ where $Xia$ is the value of trait i for species a, $Xib$ the value of trait i for species b, and $max(Xi)$ and $min(Xi)$ are, respectively, the maximum and minimum values of trait *i* for all the species considered. For all metrics, we used a total of nine common functional traits (N=9): leaf length, leaf width, maximum plant height, vegetative plant height, seed dry biomass, specific leaf area, leaf dry-matter content, rooting depth and root diameter (Table S1). In order to estimate an average dissimilarity value (Table S2; Fig, S1) from index values of comparable order of magnitude, Euclidean metrics were divided by 1000 in case of unstandardized data and by 10 when standardized.

Table S1. Description, units, mean value (and standard deviation), and references for the common traits used to calculate the pairwise functional dissimilarity between the three target species.

| **Trait** | **Units** | ***S.tenacissima*** | ***B. retusum*** | ***L. spartum*** | **References** |
| --- | --- | --- | --- | --- | --- |
| Leaf length | mm | 66.67(7.64) | 8.68(2.64) | 44.50(12.68) | [5, 6, 7, 8, 9, 10, 26] |
| Leaf width | mm | 2.25(0.35) | 2.56(0.62) | 1.33(0.14) | [5, 6, 11, 12, 13] |
| Max. height | cm | 151.25(22.50) | 48.33(2.89) | 75.00(8.00) | [5, 9, 12, 13, 14, 15] |
| Plant height | cm | 89.25(27.91) | 29.23(10.10) | 61.60(14.90) | [6, 7, 9, 16, 17, 18, 19] |
| Seed dry mass | mg | 4.94(2.09) | 2.94(0.13) | 63.35(49.29) | [16, 18, 20, 21, 22, 23] |
| SLA^1^ | cm^2^ g^-1^ | 17.40(2.45) | 123.32(75.91) | 23.60(4.37) | [7, 18, 24, 25, 26, 27] |
| LDMC^2^ | mg g^-1^ | 212.70(na) | 483.70(na) | 161.30(na) | [24, 26] |
| Rooting depth | cm | 30.00(14.14) | 17.50(3.54) | 40.00(14.14) | [6, 17, 19, 28] |
| Root diameter | mm | 1.29(0.84) | 0.85(0.69) | 2.23(1.77) | [19] |

^1^ SLA: Specific leaf area, estimated as the relation between the leaf area and the leaf mass.

^2^ LDMC: Leaf dry-matter content, calculated as the relation between leaf dry mass and leaf fresh mass.

 Table S2. Measures of pair-wise dissimilarity between the target species based on three different metrics calculated from the original, unstandardized trait data; Ln-transformed trait values (Ln); and trait values relative to the maximum value over the target species (Zmax). BR-ST, ST-LS, and BR-LS correspond to the pair-wise combinations *B. retusum - S. tenacissima*, *S. tenacissima - L. spartum,* and *B. retusum - L. spartum*, respectively.

| **Dissimilarity index** | **BR-ST** | **ST-LS** | **BR-LS** |
| --- | --- | --- | --- |
| **Gower metric** | 0.67 | 0.51 | 0.82 |
| **Gower metric (Ln)** | 0.69 | 0.46 | 0.86 |
| **Euclidean distance /1000** | 0.32 | 0.11 | 0.35 |
| **Euclidean distance (Ln) /10** | 0.35 | 0.28 | 0.44 |
| **Euclidean distance (Zmax)/10** | 0.17 | 0.13 | 0.19 |
| **Bray-Curtis index** | 0.47 | 0.26 | 0.51 |
| **Bray-Curtis index (Ln)** | 0.15 | 0.10 | 0.19 |
| **Bray-Curtis index (Zmax)** | 0.41 | 0.27 | 0.49 |
| **Average (±SE)*** | **0.41 (±0.10)** | **0.29 (±0.07)** | **0.50 (±0.12)** |

* Average values calculated only from the metrics based on standardized trait data (Ln and Zmax)


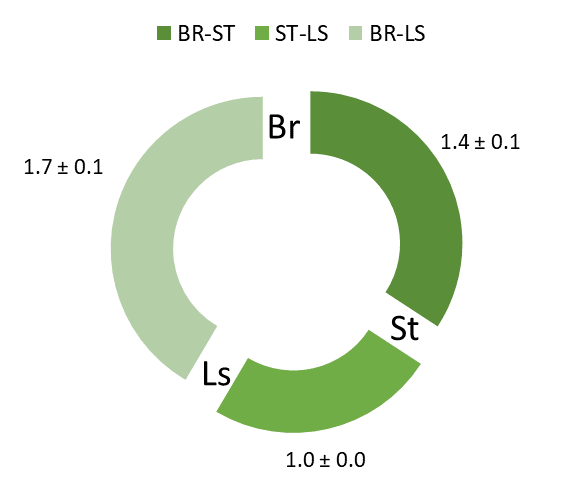


Figure S1. Average (±SE) relative dissimilarity between the target species. Values based on standardized trait data and from metric values expressed relative to the minimum pairwise distance (ST-LS in all cases). BR-ST, ST-LS, and BR-LS correspond to the pair-wise combinations *B. retusum - S. tenacissima*, *S. tenacissima - L. spartum,* and *B. retusum - L. spartum*, respectively.

**References**

1. Sokal RR, Sneath PHA. Principles of numerical taxonomy. Witt. Freeman and Co., San Francisco. 1963.
2. Sokal, R. R. & Michener, C. D., 1957. The effects of different numerical techniques on the phenetic classification of bees of the *Hoplitis* complex (*Megachilidae*). Proc. Linn Soc. London 178: 59-74.
3. Bray JR, CuBray JR, Curtis JT. An ordination of the upland forest communities in southern Wisconsin. Ecol Monogr. 1957; 27: 325-349.
4. Faith DP, Minchin PR, Belbin L. Compositional dissimilarity as a robust measure of ecological distance. Vegetatio. 1982; 69: 57-68.
5. Clayton WD, Vorontsova MS, Harman KT, Williamson H. GrassBase - The Online World Grass Flora. 2006; http://www.kew.org/data/grasses-db.html (Accessed February 2019).
6. Caturla RN, Raventós J, Guàrdia R, Vallejo VR. Early post-fire regeneration dynamics of *Brachypodium retusum* Pers. (Beauv.) in old fields of the Valencia region (eastern Spain). Acta Oecol. 2000; 21: 1-12.
7. Pugnaire FI, Haase P. Comparative physiology and leaf growth of two perennial tussock grass species in a semi-arid environment. AoB Plants. 1996; 77: 81-86.
8. Cope T, Nesbitt M. *Lygeum spartum*. Curtis's Botanical Magazine. 2002; 19: 35-39.
9. Mateo G, Crespo MB. Claves para la flora valenciana. 1990; Ed. Del Cenia al Segura.
10. Pajarón, S Escudero A. Guía botánica de las Sierras de Cazorla, Segura y Alcaraz. Editorial Pirámide. 1993.
11. Rejos FJ. La Atocha (*Stipa tenacissima* Loefl. ex L.) en el centro peninsular: aspectos vegetativos y reproductivos. Tesis Doctoral, Universidad de Alcalá. 2000.
12. Bolunin O. Guía de campo de las flores de Europa. Ed.Omega. 1982.
13. *Lygeum spartum* L. Gen. Pl., ed. 5. [522]. 1754
14. Kattge J, Díaz S, Lavorel S, Prentice IC, Leadley P, Bönisch G, … Wirth C. TRY - A global database of plant traits. Global Change Biol. 2011; 17: 2905– 2935.
15. Carreira JA, Neill FX. Plant nutrient changes in a semi-arid Mediterranean shrubland after fire. J Veg Sci. 1992; 3: 457-466.
16. Paula S, Arianoutsou M, Kazanis D, Tavsanoglu Ç, Lloret F, Buhk C, Ojeda F, Luna B, Moreno JM, Rodrigo A, Espelta JM, Palacio S, Fernández-Santos B, Fernandes PM, Pausas JG. Fire-related traits for plant species of the Mediterranean Basin. Ecol. 2009; 90: 1420.
17. Puigdefábregas J, Mendizabal T. Perspectives on desertification: western Mediterranean. J Arid Environ. 1998; 2: 209-224.
18. Frenette-Dussault C, Shipley B, Léger JF, Meziane D, Hingrat Y. Functional structure of an arid steppe plant community reveals similarities with Grime's C-S-R theory. J Veg Sci. 2012; 23: 208-222.
19. De Baets S, Poesen J, Knapen A, Barberá GG, Navarro JA. Root characteristics of representative Mediterranean plant species and their erosion-reducing potential during concentrated runoff. Plant Soil. 2007; 294: 169–183.
20. Royal Botanical Gardens KEW. Seed Information Database (SID), http://data.kew.org/sid/ (Accessed February 2019).
21. Seed Bank, Valencia Regional Government. Spain.
22. Haase P, Pugnaire FI, Incoll LD. Seed production and dispersal in the semi-arid tussock grass *Stipa tenacissima* L. during masting. J Arid Environ. 1995; 31: 55-65.
23. Cerdá A, García-Fayos P. The influence of slope angle on sediment, water and seed losses on badland landscapes. Geomorphology. 1997; 18: 77-90.
24. Ghiloufi W, Chaieb M. Effect of biological soil crusts on soil chemical properties: a study from Tunisian arid ecosystem. International Journal of Agronomy and Agricultural Research (IJAAR). 2014; 4: 22-32.
25. Garnier E, Cordonnier P, Guillerm JL, Sonié L. Specific leaf area and leaf nitrogen concentration in annual and perennial grass species growing in Mediterranean old-fields. Oecol. 1997; 111: 490-498.
26. Bautista S, Turrión D, Mayor AG. (unpublished data)
27. Vega S, Nazarova V, Bladé C, Bautista S. (unpublished data)
28. Nedjimi B. *Lygeum spartum* L.: a review of a candidate for West Mediterranean arid rangeland rehabilitation. Rangeland J. 2016; 38: 493-499.
